# R&D and pricing strategies in the intelligent vehicle supply chain under cross-organizational cooperative models

**DOI:** 10.1371/journal.pone.0321903

**Published:** 2025-04-24

**Authors:** Hui Yu, Fei Song

**Affiliations:** School of Economics and Business Administration, Chongqing University, Chongqing, China; Czestochowa University of Technology: Politechnika Czestochowska, Poland

## Abstract

With the development of automotive intelligence, vehicle manufacturers and technology companies have engaged in cross-industry cooperative research and development (R&D), leading to new cooperative R&D models. In this context, this paper develops game-theoretic models for four distinct cooperative frameworks: the technology-dominant model, the supplier-cooperation model, the manufacturer-dominant model, and the joint decision-making model. This paper aims to explore the conditions under which different cooperative R&D models are applicable and examines the strategic choices associated with each model. The results indicate that if a company is a core industry player, the core component supplier should select the technology-dominant model, while the vehicle manufacturer should choose the manufacturer-dominant model. If it is not a core player, the joint decision-making model is the next best choice. If the goal of R&D cooperation is achieving a high level of intelligence rather than short-term profits, the manufacturer-dominant model is more suitable. Though price wars may emerge, under the technology-dominant model, the supply chain adopts a quality strategy. Across the other three models, quality strategies remain consistent. As the consumer price sensitivity coefficient increases, the supply chain is more likely to adopt both quality and low-price strategies simultaneously. These findings offer valuable insights for making R&D and pricing strategy decisions under cross-organizational cooperative models in the intelligent vehicle supply chain.

## 1 Introduction

Advancements in Artificial Intelligence (AI), particularly in the development of large-scale models, have propelled autonomous driving systems from simple automation to highly intelligent systems capable of autonomous perception and decision-making [1, 2]. This shift has propelled the vehicle supply chain into an era of intelligent competition, where vehicle intelligence has become a pivotal factor influencing consumer decision-making. Vehicle manufacturers are advancing technological innovations to capitalize on market opportunities. According to Grand View Research, the global intelligent transportation market is projected to grow at a compound annual growth rate of 13.0% between 2023 and 2030 [3]. J.D. Power revealed that the influence of intelligent features on purchasing decisions rose from 12% in 2022 to 14% in 2023, making it the third most important factor after vehicle quality and performance [4]. Additionally, the advancement of vehicle intelligence necessitates continuous and substantial investment in research and development (R&D). Therefore, both technology companies and vehicle manufacturers have already committed considerable resources. For example, Waymo has invested over USD 3 billion in autonomous driving technology, focusing on areas such as sensor development and software optimization. Similarly, Tesla’s R&D expenditure reached USD 4.8 billion in 2022, with a substantial portion dedicated to enhancing its autonomous systems. Furthermore, General Motors has committed more than USD 27 billion toward intelligent vehicle development through 2025, concentrating on integrating advanced driver-assistance systems and fully autonomous capabilities across its vehicle lineup.

In response to the substantial investments required for developing intelligent technologies, particularly autonomous driving systems, companies with varying strengths are engaging in cross-organizational R&D cooperation [5–8], especially between technology firms and vehicle enterprises. At this point, both the power structure and cooperation models in the supply chain have undergone changes. For instance, Huawei has proposed three cooperation models with automotive companies: the Huawei Smart Selection model, the Huawei Inside model, and the Component Supplier model. Similarly, Google, through its subsidiary Waymo, cooperates with various vehicle manufacturers to develop autonomous driving systems. In cooperation on R&D in the intelligent vehicle supply chain, two key trends have emerged: heightened competition in R&D and aggressive pricing strategies [9]. To maintain technological leadership and meet evolving market demands, vehicle manufacturers have significantly increased their R&D investments. For instance, Huawei’s R&D expenditure reached RMB 161.5 billion in 2022, representing a quarter of its annual revenue. BYD’s R&D spending increased by 90.31% year-on-year to RMB 20.223 billion. Other major vehicle manufacturers, such as General Motors, Daimler, and Ford, have committed billions of dollars to intelligent vehicle R&D and production over the next few years. On the pricing front, competition in the 2024 vehicle market has intensified. For instance, a report from CnEVPost reveals that a total of 195 models saw price cuts between January and September 2024, surpassing the total number of price reductions recorded in all of 2023 in China. These reductions have been part of an intense price war [10]. This wave of price reductions was initiated by Tesla’s 6% price cut for its Model 3 and Model Y vehicles in China, prompting similar actions from BYD, NIO, XPeng, and others to maintain market share. Therefore, studying the strategic choices in the context of cooperative R&D models in the intelligent vehicle supply chain holds significant academic value and practical relevance.

This paper aims to address the following critical questions: How should technology companies and vehicle manufacturers select cooperative R&D model? What strategy should be chosen under different cross-organizational cooperation models? Specifically, should a low-price strategy, a quality strategy, or a combination of both be adopted? These questions represent urgent issues for the intelligent vehicle supply chain. By analyzing the impact of process innovation, product innovation, and R&D cost-sharing on R&D effort, pricing decisions, and the profits of each entity, this paper makes the following contributions: Firstly, this paper identifies the conditions under which each model applies and examines changes in power structures across various cooperative R&D models. Secondly, by analyzing the primary factors influencing R&D efforts and pricing decisions in the intelligent vehicle supply chain, this paper clarifies the strategic options available under different cooperation models.

The structure of this paper is as follows: Section 2 reviews the relevant literature. Section 3 describes the research problem and outlines key assumptions. Section 4 develops four cooperative R&D models in the supply chain, deriving the optimal R&D effort level, vehicle pricing, and profit for each participant. Section 5 offers a comparative analysis of equilibrium outcomes across models, examining the effects of process innovation, product innovation, and R&D cost-sharing, and identifying conditions under which each model and strategy applies. Section 6 presents numerical analysis, and Section 7 concludes with key findings, managerial implications, and suggestions for future research.

## 2 Literature review

With the rapid expansion of the intelligent vehicle market, studying cooperation models in the intelligent vehicle supply chain has emerged as a critical issue. This paper is primarily related to three streams of literature: cross-organizational joint R&D, supply chain power structures, and the vehicle supply chain.

Cross-organizational cooperative innovation is a key strategy for companies to achieve technological advancement and gain competitive advantages [7,8,11]. Initially, studies concentrated on R&D cooperation in the supply chain through technology licensing. For instance, Wu and Kao [12] established a cooperative framework between original equipment manufacturers (OEMs) and independent remanufacturers, involving technology licensing and joint R&D ventures. Zhang et al. [13] investigated technology licensing between OEMs and contract manufacturers, while Yan et al. [11] examined the effects of different licensing models on inter-firm R&D cooperation. As market demands and technological complexity have grown, scholars have turned their attention to more intensive R&D cooperation in supply chains, with a particular emphasis on the research of general purpose technology. General purpose technology development frequently requires cross-industry R&D cooperation since these technologies embody both public and proprietary characteristics. Several studies have explored the impact of cross-organizational R&D on common technologies from various angles, including knowledge spillovers, incentive contracts, and bilateral cooperation [6-9, 14]. These studies highlight how companies share technological outcomes and risks through joint R&D. Additionally, scholars have examined other impacts of cross-organizational R&D in supply chains. Zhou et al. [15] explored cooperative innovation decisions in innovation cartels and research joint ventures. Gupta et al. [16] investigated value co-creation models from a multi-supplier procurement perspective. Guo et al. [17] proposed a theoretical framework for R&D alliances using complex network evolutionary games. Zhang et al. [8] analyzed the effects of government subsidies on OEM and IR cooperation decisions. Wang et al. [18] studied cooperative competition in the new energy vehicle supply chain under R&D subsidies. Wei et al. [19] explored manufacturers’ optimal strategy choices among independent innovation, cooperation with existing suppliers, or partnerships with new suppliers. Liu et al. [20] examined cooperative contracts between competitors. Notably, Eklund [5] examined the impact of centralized and decentralized power on the quantity and quality of innovation R&D. However, the study was confined to internal organizational settings and did not explore supply chain cooperation.

Studies on supply chain power structures primarily focus on three types: manufacturer-dominated, retailer-dominated, and vertical Nash [21–29]. In the manufacturer-dominated structure, the manufacturer holds a dominant position in the supply chain, and its decisions influence those of the retailer; in the retailer-dominated structure, the retailer dominates the supply chain, with its decisions affecting the manufacturer’s decisions; in the Vertical Nash structure, power within the supply chain is relatively balanced, with manufacturers and retailers collaborating through game theory to jointly formulate strategies that optimize overall supply chain benefits. Specifically, Li and Mizuno [21] examined periodic review, joint dynamic pricing, and inventory management in a dual-channel supply chain with random demand and price-sensitive manufacturers and retailers. Chen et al. [22] explored how product substitutability affects firm performance. Hu et al. [23] analyzed supply chain preferences under both symmetric and asymmetric information structures. Jena and Meena [24] constructed four types of supply chain power structures: manufacturer-dominated, retailer-dominated, vertical Nash, and cooperative, and examined price competition between manufacturers and remanufacturers. Zhang et al. [25] investigated how wholesale price and cost-sharing contracts impact product greenness and demand under consumer reference price effects. Zhang et al. [26] studied green advertising investments and pricing decisions in the fashion industry under various power structures between manufacturers and leasing platforms. Fu et al. [27] explored how different power structures influence group buying among competing firms. In the financial sector, Li et al. [28] analyzed the application of agricultural insurance, constructing insurer-dominated, bank-dominated, and vertical Nash structures. Hu [29] examined the relationship between capital structure and supply chain capacity. Subsequent studies have explored supply chain power structures with multiple stakeholders. Hu et al. [30] examined how the acquisition of first-tier suppliers affects supply chain structure and its impact on core firms’ selling costs. Gu et al. [31] explored the role of regret in retailer decision-making in competitive multi-stakeholder supply chains. Wang and Zhu [32] analyzed a supply chain involving a government, hub port, and two carriers, focusing on the impact of carbon tax policies on competition and cooperation incentives. Qiu et al. [33] proposed three power structures, namely, dominance of online retail platforms, parity between second-hand and retail platforms, and dominance of second-hand platforms. They also studied the effectiveness of vertical integration between recycling and second-hand platforms.

Studies on vehicle supply chain management primarily focus on the impacts of subsidies and low-carbon policies, supply chain optimization, and vehicle trade-in programs. Regarding the influence of vehicle policies on supply chain operational decisions, Cheng et al. [34] examined how price threshold subsidies incentivize sustainable operations for vehicle companies. Liu et al. [35] investigated optimal pricing strategies for vehicle supply chain members under dual policies, considering consumers’ low-carbon preferences. Wang et al. [36] provided theoretical foundations and strategic guidance for vehicle manufacturers to cope with remanufacturing competition under carbon tax policies. Feng et al. [37] explored how carbon reduction measures affect the adoption of blockchain technology in the new energy vehicle industry. Research on vehicle supply chain efficiency optimization. Tu et al. [38] applied graph neural networks to analyze graph-structured data in supply chains, effectively identifying alternative suppliers during disruptions. Ji et al. [39] developed a bi-objective mixed-integer model and iterative algorithm to explore how vehicle manufacturers can leverage the Physical Internet (PI) to achieve a digitalized supply chain. Sarkar et al. [40] created a prediction-based dynamic multi-objective optimization algorithm to help the vehicle industry achieve sustainability across environmental, economic, and social dimensions. Manimuthu et al. [41] proposed an automated decision-making framework combining federated learning and smart contracts to optimize resource allocation and process efficiency in the vehicle industry. Research on Vehicle Trade-In Programs and Other Studies. Saunders et al. [42] explored how used car retailers can increase sales by managing product variety and analyzed the relationship between variety and sales. Hu et al. [43] examined when manufacturers should offer trade-in (and refurbishment) programs for strategic consumers. Additionally, other studies addressed various topics. Pu [44] analyzed the impact of equity strategies in the new energy vehicle supply chain on corporate and policy decisions. Meanwhile, Sun et al. [45] reviewed the optimization of vehicle distribution to dealerships over the past 30 years and proposed future research directions to bridge the gap between industry practices and academic research.

In conclusion, although vehicle supply chain management has been widely studied, as shown in Table 1, certain critical aspects of the intelligent vehicle supply chain remain underexplored. These include the high costs of R&D, shifts in power structures, and strategic choices influenced by different power dynamics. Additionally, the current literature offers only limited insight into how supply chain power structures affect R&D cooperation, often addressing only one aspect of R&D innovation. To fill these research gaps, this paper makes the following academic contributions. First, four supply chain power structures are proposed based on real-world scenarios in the intelligent vehicle industry, considering the different decision-making roles in R&D efforts and vehicle pricing between core component suppliers and vehicle manufacturers. Second, this paper analyzes shifts in power dynamics across various cooperative R&D models in the supply chain, providing guidance on selecting appropriate cooperation models and strategies. Third, in terms of model construction, this paper classifies R&D innovation cooperation into two dimensions: process innovation, which reduces production costs, and product innovation, which enhances product intelligence. It further examines the impact of process innovation coefficients, product innovation coefficients, and R&D cost-sharing ratios on the optimal R&D effort level, vehicle price, and the optimal profit for each participant. These insights help explain cross-industry open innovation between technology and vehicle companies and contribute to the research on cross-organizational cooperative R&D innovation in supply chains.

**Table 1:** Positioning of this paper in the literature.

Papers	Cooperative R&D effort	Supply chain power structure	Strategic choice	Process and product innovation
**Wu and Kao (2018); Yan et al (2023); Zhang et al. (2023)**	√			
**Li and Mizuno (2022); Chen et al (2024); Hu et al. (2024)**		√		
**Zheng et al. (2023); Zheng et al. (2024)**	√	√		
**Eklund (2022); Wei et al. (2024)**	√		√	
**Gupta (2023)**			√	
**Jena and Meena (2022); Zhang et al. (2022); Qiu et al. (2023)**		√	√	
**This paper**	√	√	√	√

## 3 Problem description and assumption

### 3.1 Research problem description

By examining current cross-industry cooperative R&D models for intelligent vehicles globally, this paper proposes four cooperation models based on the core enterprises in the supply chain (as shown in Table 2). These include the technology-dominant, supplier-cooperation, manufacturer-dominant, and joint decision-making models, each reflecting different power dynamics and strategic choices in real-world cooperation between core component suppliers and vehicle manufacturers. The technology-dominant model arises when core suppliers like Huawei or Waymo lead innovation and control both R&D and pricing, typically in markets driven by rapid technological advancements. In the supplier-cooperation model, suppliers such as CATL (with BMW) provide key technologies, but vehicle manufacturers retain pricing control to adjust for market demand. The manufacturer-dominant model occurs when vehicle manufacturers like Tesla or XPeng, with strong in-house R&D capabilities and market influence, oversee both innovation and pricing. Finally, the joint decision-making model is seen in highly integrated partnerships (e.g., Honda-GM), where suppliers and manufacturers share R&D and pricing responsibilities to address complex market needs.

**Table 2:** Classification of cooperation modes.

Model	Model characteristics	Enterprise cooperation
Technology-dominant model	Core component suppliers determine the R&D effort and the vehicle price.	(1) Huawei intelligent selection model. (2) Baidu Apollo’s cooperation with various car companies. (3) Aptiv PLC and Hyundai cooperation.
Supplier-cooperation model	Core component suppliers determine the R&D effort, and vehicle manufacturers determine the vehicle price.	(1) Huawei HI model. (2) Cooperation between BMW and Ningde Era (CATL). (3) Cooperation between LG Chem and General Motors.
Manufacturer-dominant model	The vehicle manufacturer determines the R&D effort and the vehicle price.	(1) Huawei’s component supply model. (2) BYD, NIO, XPeng, Xiaomi, Toyota.
Joint decision-making model	Jointly determine the R&D effort and the vehicle price.	(1) Cooperation between Honda and GM. (2) Renault-Nissan-Mitsubishi alliance. (3) Volkswagen and Ford’s joint investment in Argo AI.

This paper first derives the optimal decisions and profit functions for each supply chain participant under the four cooperation models. It then compares the optimal profit, R&D effort, vehicle pricing decisions, and overall supply chain profits across different models, analyzing the influence of key parameters on the equilibrium results. The notations and descriptions used in this paper are shown in Table 3.

**Table 3:** Notations and descriptions.

Notations	Descriptions
*i*	Superscript. Supply chain participants, i=Z,S,SC refers to vehicle manufacturers, core component suppliers, and the entire supply chain.
*j*	Subscript. Cooperation models, j=SH,SZ,ZH,LH refers to technology-dominant, supplier cooperation, manufacturer-dominant, and joint decision-making models.
*a*	Potential market demand for intelligent cars
*b*	Consumer price sensitivity coefficient
*β*	Sensitivity coefficient of consumer product quality
*θ*	Research and development to reduce cost sensitivity
*h*	Cost factor of R&D investment, h>0
*g*	Component costs of core component suppliers
$c_{0}$	Vehicle manufacturer’s initial production cost, $\tilde{c}<c_{0}$
$\tilde{c}$	Vehicle manufacturer production cost floor, $0<\tilde{c}$
*τ*	Percentage of R&D cost shared by core component suppliers. τ=1 indicates independent research and development of core component suppliers, τ=0 indicates independent research and development of vehicle manufacturers, $\tau \in \left(0,1\right)$.
$p_{0}$	Vehicle manufacturer’s lowest price
*w*	Wholesale price of parts
*p*	intelligent car price (vehicle price), $p\geq p_{0}$
*e*	R&D development level of intelligent vehicles
$\prod_{j}^{i}$	*i* ‘s profit in model *j*
$C\left(e\right)$	Vehicle manufacturer cost function, $C\left(e\right)=c_{0}-\theta e$.
$T\left(e\right)$	Technical service fees charged by core component suppliers: $T\left(e\right)=t_{0}+ke$. Where *k* is the unit R&D efforts technical service fee, $t_{0}$ is a fixed service fee.

### 3.2 Fundamental assumptions

**Fig 1:**
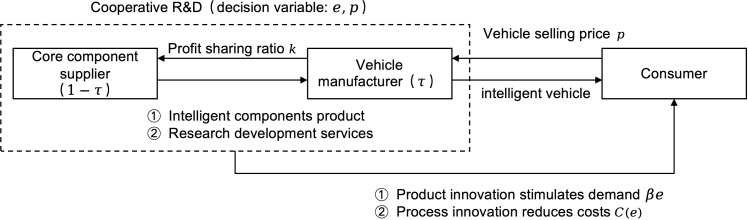
Supply chain cooperative research and development model.

Assume there is a risk-neutral technology firm serving as the core component supplier, primarily responsible for providing intelligent vehicle technologies and components such as autonomous driving systems, intelligent connectivity solutions, and battery management systems. Meanwhile, the vehicle manufacturer is responsible for vehicle manufacturing, including body design, assembly, and quality control. To keep pace with the rapid evolution of vehicle intelligence, the core component supplier and the vehicle manufacturer engage in cooperative R&D to enhance their competitiveness. In this cooperation, the vehicle manufacturer not only procures intelligent components but also receives technical R&D support from the core component supplier. Furthermore, both parties share R&D costs to foster continuous innovation and accelerate the application of advanced technologies [46]. This cooperative R&D not only leads to product or service quality improvements but also reduces production costs. For example, in the cooperation between Huawei and Seres, they not only jointly established the Seres Super Factory but also cooperated in developing the AITO series, enhancing both production efficiency and product intelligence. The above is shown in Fig 1.

Based on the above description and to simplify the game decision-making model for cooperative R&D, we propose the following basic assumptions:

(1) Unit production cost of a vehicle manufacturing enterprise C is a function of the R&D effort e, specifically $C\left(e\right)=c_{0}-\theta e$. Where $c_{0}$ is the initial production cost, *θ* is the sensitivity factor of R&D cost reduction. A higher *θ* indicates a higher degree of cost reduction due to R&D efforts. However, there is a lower bound to the cost reduction, that is, there exists a minimum material consumption cost denoted as $\tilde{\text{c}}$. In summary, the production cost satisfies the following condition, $\tilde{c}<C\left(e\right)\leq c_{0}$, $0\leq e<\left(c_{0}-\tilde{c}\right)/\theta$.
(2) The automobile demand function is D=a−bp+βe. Where *β* represents the sensitivity coefficient of consumer product quality, and the higher the *β*, the higher the degree of increase of R&D efforts on demand [6].
(3) According to the research of Zheng YL et al. [6] and the real situation, the technical service fee charged by the core component supplier T is a function of the R&D effort e, specifically $T\left(e\right)=t_{0}+ke$. Where *k* is the unit research and development efforts technical service fee, $t_{0}$ is a fixed service fee.
(4) With reference to the studies of Atasu and Subramanian [47] and Ranjan and Jha [48], it is assumed that the cost of R&D investment is a quadratic function of R&D investment level, i.e., $h{\text{e}}^{\text{2}}/2$. Where, *e* represents the level of R&D effort of the enterprise, *h* represents the cost coefficient of R&D effort of the enterprise, and the smaller *h* is, the higher the R&D efficiency.

## 4 Model construction and solution

This paper first determines the R&D effort level before setting the vehicle price, reflecting the prioritization of innovation over pricing in response to intelligent competition. By focusing on R&D efforts first, companies can establish a competitive edge through technological advancements, which subsequently informs their pricing strategy. Based on industry trends, enterprises can adopt either a quality strategy or a low-price strategy. A quality strategy emphasizes high-quality products to build brand image and market competitiveness, while a low-price strategy focuses on reducing prices to capture market share. Furthermore, the four power structure models presented in this paper constitute a basic assumption in the study of supply chain power structures. This paper not only encompasses the three common power structures [21–29], but also introduces a joint decision-making power structure similar to that proposed by Jena and Meena [24]. The construction of the specific profit function is similar to that in existing literature [6, 7]. However, unlike existing research in the field of power structures, the four models developed in this paper involve not only a change in the decision-making sequence but also a shift in the allocation of decision-making authority, which represents the innovation of this paper.

### 4.1 Technology-dominant model

In this model, the core component supplier determines the R&D effort $e_{SH}$ and sets the vehicle price $p_{SH}$, while the vehicle manufacturer will only agree to cooperate if its profit is non-negative. Consequently, it is assumed that the vehicle manufacturer will offer the core component supplier a minimum price $p_{0}$. In this scenario, the core component supplier becomes the dominant entity in the supply chain, relegating the vehicle manufacturer to a lower position, effectively serving as a contract manufacturer. For example, in the ‘Huawei Smart Selection’ cooperative R&D model between Huawei and Seres, Huawei leads the design, sales, production, and services for the AITO intelligent car brand. Both the R&D effort level and vehicle pricing for AITO are determined by Huawei. The assumptions in the subsequent sections follow the same logic as those in this section. Similar to previous literature [6,7], the profit function of the core component supplier can be expressed as:


$$\prod_{SH}^{S}\left(e_{SH},p_{SH}\right)=\left(w-g\right)\left(a-bp_{SH}+\beta e_{SH}\right)+T\left(e_{SH}\right)-\tau he_{SH}^{2}/2$$ (1)

The profit function of the vehicle manufacturer is:


$$\prod_{SH}^{Z}=\left(p_{SH}-w-C\left(e_{SH}\right)\right)\left(a-bp_{SH}+\beta e_{SH}\right)-T\left(e_{SH}\right)-\left(1-\tau \right)he_{SH}^{2}/2$$ (2)

The objective function of the core component supplier is $\underset{e_{SH},p_{SH}}{Max}\prod_{SH}^{S}\left(e_{SH},p_{SH}\right)$.

Taking the first derivative of equation (1) with respect to $p_{SH}$ gives: $\partial \prod_{SH}^{S}\left(e_{SH},p_{SH}\right)/\partial p_{SH}=-b\left(w-g\right)<0$, which indicates that the profit function of the core component supplier decreases with increasing price $p_{SH}$. Consequently, the core component supplier will set the price at the base price $p_{0}$, as provided by the vehicle manufacturer, so:


$$p_{SH}^{*}=p_{0}$$ (3)

Substitute equation (3) into equation (1), we can get:


$$\prod_{SH}^{S}\left(e_{SH},{\text{p}}_{SH}\right)=\left(w-g\right)\left(a-bp_{0}+\beta e_{SH}\right)+T\left(e_{SH}\right)-\tau he_{SH}^{2}/2$$ (4)

Then, we can find the first derivatives of equation (4) with respect to ${\text{e}}_{SH}$:


$$\partial \prod_{SH}^{S}\left(e_{SH},p_{SH}\right)/\partial e_{SH}=\beta \left(w-g\right)+k-\tau he_{SH}$$ (5)

And the second derivatives of equation (4) with respect to ${\text{e}}_{SH}$ is:


$$\partial^{2}\prod_{SH}^{S}\left(e_{SH},p_{SH}\right)/\partial e_{SH}^{2}=-\tau h<0$$ (6)

According to equation (6), $\prod_{SZ}^{Z}\left(p_{SZ}\right)$ reaches a maximum, which occurs at $\partial \prod_{SH}^{S}\left(e_{SH},p_{SH}\right)/\partial e_{SH}=0$. Setting equation (5) equals to 0, the optimal R&D effort level under the technology-dominant model can be obtained as:


$$e_{SH}^{*}=\left(\beta \left(w-g\right)+k\right)/\tau h$$ (7)

By substituting equations (3) and (7) into (1), we can obtain the optimal profit of the core component supplier under the technology-dominant model:


$$\prod_{SH}^{S*}\left(e_{SH},p_{SH}\right)=\left(w-g\right)\left(a-bp_{0}+2\beta A\right)+t_{0}+2kA-2\tau hA^{2}$$ (8)

By substituting equations (3) and (7) into (2), we can obtain the optimal profit of the vehicle manufacturer under the technology-dominant model:


$$\prod_{SH}^{Z*}=\left(p_{0}-w-c_{0}+2\theta A\right)\left(a-bp_{0}+2\beta A\right)-t_{0}-2kA-\left(1-\tau \right)2hA^{2}$$ (9)

Where $A=\left(\beta \left(w-g\right)+k\right)/2\tau h$.

**Theorem 1:** Under the technology-dominant model.

The optimal vehicle selling price is: $p_{SH}^{*}=p_{0}$.

The optimal R&D effort level is: $e_{SH}^{*}=\left(\beta \left(w-g\right)+k\right)/\tau h$.

The profit of core component supplier is:


$$\prod_{SH}^{S*}\left(e_{SH},p_{SH}\right)=\left(w-g\right)\left(a-bp_{0}+2\beta A\right)+t_{0}+2kA-2\tau hA^{2}.$$

The profit of the vehicle manufacturer is:


$$\prod_{SH}^{Z*}=\left(p_{0}-w-c_{0}+2\theta A\right)\left(a-bp_{0}+2\beta A\right)-t_{0}-2kA-\left(1-\tau \right)2hA^{2}.$$

### 4.2 Supplier-cooperation model

In the supplier-cooperation model, the core component supplier first decides the R&D effort $e_{SZ}$, and the vehicle manufacturer subsequently determines the vehicle pricing $p_{SZ}$. In this model, the core component supplier and the vehicle manufacturer hold relatively equal positions in the supply chain. For instance, in the cooperative R&D between Huawei and Changan, Huawei leads the R&D efforts, while Changan retains control over vehicle pricing for the Avatr cooperative model. The profit function of the core component supplier is:


$$\prod_{SZ}^{S}\left(e_{SZ}\right)=\left(w-g\right)\left(a-bp_{SZ}+\beta e_{SZ}\right)+T\left(e_{SZ}\right)-\tau he_{SZ}^{2}/2$$ (10)

The profit function of the vehicle manufacturer is:


$$\prod_{SZ}^{Z}\left(p_{SZ}\right)=\left(p_{SZ}-w-C\left(e_{SZ}\right)\right)\left(a-bp_{SZ}+\beta e_{SZ}\right)-T\left(e_{SZ}\right)-\left(1-\tau \right)he_{SZ}^{2}/2$$ (11)

The objective function of the core component supplier is $\underset{e_{SZ}}{Max}\prod_{SZ}^{S}\left(e_{SZ}\right)$ and the objective function of the vehicle manufacturer is $\underset{p_{SZ}}{Max}\prod_{SZ}^{Z}\left(p_{SZ}\right)$.

Backward induction is used to solve, and we can find the first derivatives of equation (11) with respect to $p_{SZ}$:


$$\partial \prod_{SZ}^{Z}\left(p_{SZ}\right)/\partial p_{SZ}=a-2bp_{SZ}+\beta e_{SZ}+bc_{0}+bw-b\theta e_{SZ}$$ (12)

And the second derivatives of equation (11) with respect to $p_{SZ}$ is:


$$\partial^{2}\prod_{SZ}^{Z}\left(p_{SZ}\right)/\partial p_{SZ}^{2}=-2b<0$$ (13)

According to equation (13), $\prod_{SZ}^{Z}\left(p_{SZ}\right)$ reaches a maximum, which occurs at $\prod_{SZ}^{Z}\left(p_{SZ}\right)/\partial p_{SZ}=0$. By setting equation (12) equals to 0, we obtain:


$$p_{SZ}=\left(a+\beta e_{SZ}+bc_{0}+bw-b\theta e_{SZ}\right)/2b$$ (14)

By substituting equation (14) into the profit function (10), we obtain:


$$\prod_{SZ}^{S}\left(e_{SZ}\right)=\left(w-g\right)\left(a+\beta e_{SZ}-bc_{0}-bw+b\theta e_{SZ}\right)/2+t_{0}+ke_{SZ}-\tau he_{SZ}^{2}/2$$ (15)

Then, we can find the first derivatives of equation (15) with respect to ${\text{e}}_{SZ}$:


$$\partial \prod_{SZ}^{s}\left(e_{SZ}\right)/\partial e_{SZ}=\left(w-g\right)\left(b\theta +\beta \right)/2+k-\tau he_{SZ}$$ (16)

And the second derivatives of equation (15) with respect to ${\text{e}}_{SZ}$ is:


$$\partial^{2}\prod_{SZ}^{S}\left(e_{SZ}\right)/\partial e_{SZ}^{2}=-\tau h<0$$ (17)

According to equation (17), $\prod_{SZ}^{S}\left(e_{SZ}\right)$ reaches a maximum, which occurs at $\partial \prod_{SZ}^{S}\left(e_{SZ}\right)/\partial e_{SZ}=0$. By setting equation (16) equals to 0, we obtain:


$$e_{SZ}^{*}=\left(\left(w-g\right)\left(\beta +b\theta \right)+2k\right)/2\tau h$$ (18)

By substituting equation (18) into (14), we obtain:


$$p_{SZ}^{*}=\left(a+bc_{0}+bw\right)/2b+\left(\beta -b\theta \right)\left(\left(w-g\right)\left(\beta +b\theta \right)+2k\right)/4b\tau h$$ (19)

By substituting equation (18) and (19) into equation (10), we can obtain the optimal profit of the core component suppliers under the supplier-cooperation model:


$$\prod_{SZ}^{S*}\left(e_{SZ}\right)=\left(w-g\right)\left(\left(a-bc_{0}-bw\right)/2+\left(\beta +b\theta \right)B\right)+t_{0}+2kB-2\tau hB^{2}$$ (20)

By substituting equation (18) and (19) into equation (11), we can obtain the optimal profit of the vehicle manufacturers under the supplier-cooperation model:


$$\prod_{SZ}^{Z*}\left(p_{SZ}\right)=\left(1/b\right){\left(\left(a-bc_{0}-bw\right)/2+\left(\beta +b\theta \right)B\right)}^{2}-t_{0}-2kB-2h\left(1-\tau \right)B^{2}$$ (21)

Where $B=\left(\left(w-g\right)\left(\beta +b\theta \right)+2k\right)/4b\tau h$.

**Theorem 2:** Under the supplier-cooperation model.

The optimal vehicle selling price is:


$$p_{SZ}^{*}=\left(a+bc_{0}+bw\right)/2b+\left(\beta -b\theta \right)\left(\left(w-g\right)\left(\beta +b\theta \right)+2k\right)/4b\tau h.$$

The optimal R&D effort level is:


$$e_{SZ}^{*}=\left(\left(w-g\right)\left(\beta +b\theta \right)+2k\right)/2\tau h.$$

The profit of core component supplier is:


$$\prod_{SZ}^{S*}\left(e_{SZ}\right)=\left(w-g\right)\left(\left(a-bc_{0}-bw\right)/2+\left(\beta +b\theta \right)B\right)+t_{0}+2kB-2\tau hB^{2}.$$

The profit of the vehicle manufacturer is:


$$\prod_{SZ}^{Z*}\left(p_{SZ}\right)=\left(1/b\right){\left(\left(a-bc_{0}-bw\right)/2+\left(\beta +b\theta \right)B\right)}^{2}-t_{0}-2kB-2h\left(1-\tau \right)B^{2}.$$

### 4.3 Manufacturer-dominant model

The vehicle manufacturer decides both the R&D effort $e_{ZH}$ and the vehicle pricing $p_{ZH}$. In this model, the core component supplier functions solely as a provider, with the vehicle manufacturer holding the dominant position in the supply chain. For instance, in the cooperation between Huawei and Audi, Huawei supplies intelligent vehicle components and basic R&D services, while Audi retains full control over the R&D effort and vehicle pricing for its cars. The profit function of the core component supplier is:


$$\prod_{ZH}^{S}=\left(w-g\right)\left(a-bp_{ZH}+\beta e_{ZH}\right)+T\left(e_{ZH}\right)-\tau he_{ZH}^{2}/2$$ (22)

The profit function of the vehicle manufacturer is:


$$\prod_{ZH}^{Z}\left(e_{ZH},p_{ZH}\right)=\left(p_{ZH}-w-C\left(e_{ZH}\right)\right)\left(a-bp_{ZH}+\beta e_{ZH}\right)-T\left(e_{ZH}\right)-\left(1-\tau \right)he_{ZH}^{2}/2$$ (23)

The objective function of the vehicle manufacturer is $\underset{e_{ZH},p_{ZH}}{Max}\prod_{ZH}^{Z}\left(e_{ZH},p_{ZH}\right)$.

When the level of R&D effort $e_{ZH}$ is fixed, we can find the first derivatives of equation (23) with respect to $p_{ZH}$:


$$\partial \prod_{ZH}^{Z}\left(e_{ZH},p_{ZH}\right)/\partial p_{ZH}=\left(a-bp_{ZH}+\beta e_{ZH}\right)-b\left(p_{ZH}-w-c_{0}+\theta e_{ZH}\right)$$ (24)

And the second derivatives of equation (23) with respect to $p_{ZH}$ is:


$$\partial^{2}\prod_{ZH}^{Z}\left(e_{ZH},p_{ZH}\right)/\partial p_{ZH}^{2}=-2b<0$$ (25)

By setting equation (24) equals to 0, we obtain:


$$p_{ZH}=\left(a+bw+bc_{0}+\left(\beta -b\theta \right)e_{ZH}\right)/2b$$ (26)

By substituting equation (26) into (23), we obtain:


$$\prod_{ZH}^{Z}\left(e_{ZH},p_{ZH}\right)=b{\left(\left(a-bw-bc_{0}+\beta e_{ZH}+b\theta e_{ZH}\right)/2b\right)}^{2}-t_{0}-ke_{ZH}-\left(1-\tau \right)he_{ZH}^{2}/2$$ (27)

We can find the first derivatives of equation (27) with respect to $e_{ZH}$:


$$\partial \prod_{ZH}^{Z}\left(e_{ZH},p_{ZH}\right)/\partial e_{ZH}=\left(a-bw-bc_{0}+\beta e_{ZH}+b\theta e_{ZH}\right)\left(\beta +b\theta \right)/2b-k-\left(1-\tau \right)he_{ZH}$$ (28)

And the second derivatives of equation (27) with respect to $e_{ZH}$ is:


$$\partial^{2}\prod_{ZH}^{Z}\left(e_{ZH},p_{ZH}\right)/\partial e_{ZH}^{2}={\left(\beta +b\theta \right)}^{2}/2b-\left(1-\tau \right)h<0$$ (29)

By setting equation (28) equals to 0, we obtain the optimal R&D efforts under the manufacturer-dominant model:


$$e_{ZH}^{*}=\left(\left(a-bw-bc_{0}\right)\left(\beta +b\theta \right)-2kb\right)/\left(2bh\left(1-\tau \right)-{\left(\beta +b\theta \right)}^{2}\right)$$ (30)

Assume that $\left(a-bw-bc_{0}\right)\left(\beta +b\theta \right)>2kb$. Here, $\left(a-bw-bc_{0}\right)\left(\beta +b\theta \right)$ represents the demand positively influenced by process and product innovation driven by R&D, while kb represents the demand negatively influenced by the technical service fees paid by the vehicle manufacturer. Only when the positive demand impact exceeds the negative impact will the core component supplier and the vehicle manufacturer engage in cooperative R&D.

Additionally, when τ≠1, ${\left(\beta +b\theta \right)}^{2}<2bh\left(1-\tau \right)<2bh$. Due to the significant R&D costs of intelligent vehicles, *h* is sufficiently large to satisfy ${\left(\beta +b\theta \right)}^{2}<2bh\left(1-\tau \right)<2bh$, thus ensuring $e_{ZH}^{*}>0$.

By substituting equation (30) into (26), we obtain the optimal vehicle price under the manufacturer-dominant model:


$$\begin{array}{c} P_{ZH}^{*}=\left(a+bw+bc_{0}\right)/2b\\ +\left(\beta -b\theta \right)\left(\left(a-bw-bc_{0}\right)\left(\beta +b\theta \right)-2kb\right)/\left(4b^{2}\left(1-\tau \right)h-2b{\left(\beta +b\theta \right)}^{2}\right) \end{array}$$ (31)

By substituting equation (30) and (31) into (22), we obtain the optimal profit of the core component supplier under the manufacturer-dominant model:


$$\prod_{ZH}^{S*}=\left(w-g\right)\left(\left(a-bw-bc_{0}\right)/2+\left(\beta +b\theta \right)C\right)+t_{0}+2kC-2\tau hC^{2}$$ (32)

By substituting equation (30) and (31) into (23), we obtain the optimal profit of the vehicle manufacturer under the manufacturer-dominant model:


$$\prod_{ZH}^{Z*}\left(e_{ZH},p_{ZH}\right)=\left(1/b\right){\left(\left(a-bw-bc_{0}\right)/2+\left(\beta +\theta \right)C\right)}^{2}-t_{0}-2kC-2\left(1-\tau \right)hC^{2}$$ (33)

Where $C=\left(\left(a-bw-bc_{0}\right)\left(\beta +b\theta \right)-2kb\right)/\left(4b\left(1-\tau \right)h-2{\left(\beta +b\theta \right)}^{2}\right)$.

**Theorem 3:** Under the manufacturer-dominant model.

The optimal vehicle selling price is:


$$\begin{array}{c} P_{ZH}^{*}=\left(a+bw+bc_{0}\right)/2b\\ +\left(\beta -b\theta \right)\left(\left(a-bw-bc_{0}\right)\left(\beta +b\theta \right)-2kb\right)/\left(4b^{2}\left(1-\tau \right)h-2b{\left(\beta +b\theta \right)}^{2}\right) \end{array}$$

The optimal R&D effort level is:


$$e_{ZH}^{*}=\left(\left(a-bw-bc_{0}\right)\left(\beta +b\theta \right)-2kb\right)/\left(2bh\left(1-\tau \right)-{\left(\beta +b\theta \right)}^{2}\right).$$

The profit of core component supplier is:


$$\prod_{ZH}^{S*}=\left(w-g\right)\left(\left(a-bw-bc_{0}\right)/2+\left(\beta +b\theta \right)C\right)+t_{0}+2kC-2\tau hC^{2}.$$

The profit of the vehicle manufacturer is:


$$\prod_{ZH}^{Z*}\left(e_{ZH},p_{ZH}\right)=\left(1/b\right){\left(\left(a-bw-bc_{0}\right)/2+\left(\beta +\theta \right)C\right)}^{2}-t_{0}-2kC-2\left(1-\tau \right)hC^{2}.$$

### 4.4 Joint decision-making model

The core component supplier and the vehicle manufacturer jointly decide the R&D effort $e_{LH}$ and the vehicle price $p_{LH}$. Honda, for example, has partnered with GM to develop vehicle platforms, jointly develop technology, and jointly set prices. The profit function of the supply chain is:


$$\prod_{LH}\left(e_{LH},p_{LH}\right)=\left(p_{LH}-g-C\left(e_{LH}\right)\right)\left(a-bp_{LH}+\beta e_{LH}\right)-he_{LH}^{2}/2$$ (34)

The objective function of the supply chain is: $\underset{e_{LH},p_{LH}}{Max}\prod_{LH}\left(e_{LH},p_{LH}\right)$

When the level of R&D effort is fixed, we can find the first derivatives of equation (34) with respect to $p_{LH}$:


$$\partial \prod_{LH}\left(e_{LH},p_{LH}\right)/\partial p_{LH}=a-2bp_{LH}+\beta e_{LH}-b\theta e_{LH}+bg+bc_{0}$$ (35)

And the second derivatives of equation (34) with respect to $p_{LH}$ is:


$$\partial^{2}\prod_{LH}\left(e_{LH},p_{LH}\right)/\partial p_{LH}^{2}=-2b<0$$ (36)

By setting equation (35) equals to 0, we obtain:


$$p_{LH}=\left(a+bg+bc_{0}+\beta e_{LH}-b\theta e_{LH}\right)/2b$$ (37)

By substituting equation (37) into (34), we obtain:


$$\prod_{LH}\left(e_{LH},p_{LH}\right)={\left(a-bg-bc_{0}+\beta e_{LH}+b\theta e_{LH}\right)}^{2}/4b-he_{LH}^{2}/2$$ (38)

We can find the first derivatives of equation (38) with respect to $e_{LH}$:


$$\partial \prod_{LH}\left(e_{LH},p_{LH}\right)/\partial e_{LH}=\left(\beta +b\theta \right)\left(a-bg-bc_{0}+\beta e_{LH}+b\theta e_{LH}\right)/2b-he_{LH}$$ (39)

And the second derivatives of equation (38) with respect to $e_{LH}$ is:


$$\partial^{2}\prod_{LH}\left(e_{LH},p_{LH}\right)/\partial e_{LH}^{2}=\left({\left(\beta +b\theta \right)}^{2}-2bh\right)/2b<0$$ (40)

By setting equation (39) equals to 0, we obtain the optimal R&D efforts under the joint decision-making model:


$$e_{LH}^{*}=\left(\beta +b\theta \right)\left(a-bg-bc_{0}\right)/\left(2bh-{\left(\beta +b\theta \right)}^{2}\right)$$ (41)

By substituting (41) into (37), we obtain the optimal vehicle price under the joint decision-making model:


$$\begin{array}{c} p_{LH}^{*}=\left(a+bg+bc_{0}\right)/2b\\ +\left(\beta^{2}-b^{2}\theta^{2}\right)\left(a-bg-bc_{0}\right)/\left(4b^{2}h-2b{\left(\beta +b\theta \right)}^{2}\right) \end{array}$$ (42)

Then we can obtain the optimal profit of the core component supplier under the joint decision-making model:


$$\prod_{LH}^{S*}=\left(w-g\right)\left(\left(a-bg-bc_{0}\right)/2+\left(\beta +b\theta \right)D\right)+t_{0}+2kD-2\tau hD^{2}$$ (43)

And the optimal profit of the vehicle manufacturer under the joint decision-making model is:


$$\begin{array}{c} \prod_{LH}^{Z*}=\left(\left(a+bg-bc_{0}\right)/2b-w+\left(\beta +b\theta \right)D/b\right)\left(\left(a-bg-bc_{0}\right)/2+\left(\beta +b\theta \right)D\right)\\ -t_{0}-2kD-2\left(1-\tau \right)hD^{2} \end{array}$$ (44)

Where $D=\left(\beta +b\theta \right)\left(a-bg-bc_{0}\right)/\left(4bh-2{\left(\beta +b\theta \right)}^{2}\right)$.

**Theorem 4:** Under the joint decision-making model.

The optimal vehicle selling price is:


$$\begin{array}{c} p_{LH}^{*}=\left(a+bg+bc_{0}\right)/2b\\ +\left(\beta^{2}-b^{2}\theta^{2}\right)\left(a-bg-bc_{0}\right)/\left(4b^{2}h-2b{\left(\beta +b\theta \right)}^{2}\right) \end{array}$$

The optimal R&D effort level is: $e_{LH}^{*}=\left(\beta +b\theta \right)\left(a-bg-bc_{0}\right)/\left(2bh-{\left(\beta +b\theta \right)}^{2}\right)$.

The profit of core component supplier is:


$$\prod_{LH}^{S*}=\left(w-g\right)\left(\left(a-bg-bc_{0}\right)/2+\left(\beta +b\theta \right)D\right)+t_{0}+2kD-2\tau hD^{2}.$$

The profit of the vehicle manufacturer is:


$$\begin{array}{c} \prod_{LH}^{Z*}=\left(\left(a+bg-bc_{0}\right)/2b-w+\left(\beta +b\theta \right)D/b\right)\left(\left(a-bg-bc_{0}\right)/2+\left(\beta +b\theta \right)D\right).\\ -t_{0}-2kD-2\left(1-\tau \right)hD^{2} \end{array}$$

## 5 Model analysis

### 5.1 Optimization decision analysis of cooperative R&D model

#### Proposition 1.

An increase in both the consumer quality sensitivity coefficient and the R&D cost reduction sensitivity coefficient leads to an enhancement in the level of intelligent R&D. Conversely, except in the manufacturer-dominant model, a higher proportion of R&D costs borne by the vehicle manufacturer increases the R&D level. In the technology-dominant model, the core component supplier prioritizes product innovation, paying little attention to the impact of R&D on reducing production costs. Specifically, $\partial e_{SH}^{*}/\partial \beta >0$, $\partial e_{SZ}^{*}/\partial \beta >0$, $\partial e_{ZH}^{*}/\partial \beta >0$, $\partial e_{LH}^{*}/\partial \beta >0$; $\partial e_{SZ}^{*}/\partial \theta >0$, $\partial e_{ZH}^{*}/\partial \theta >0$, $\partial e_{LH}^{*}/\partial \theta >0$; $\partial e_{SH}^{*}/\partial \tau <0$, $\partial e_{SZ}^{*}/\partial \tau <0$, $\partial e_{ZH}^{*}/\partial \tau >0$.

Proposition 1 reveals that the R&D level in intelligent vehicles is influenced by consumer quality sensitivity, and R&D cost reduction sensitivity. Moreover, the positive or negative correlations between these factors do not change with the variation of supply chain cooperation models. As consumer sensitivity to product quality rises, so does the demand for enhanced intelligent features, prompting core component suppliers to invest more in R&D to improve performance and meet these expectations. This is exemplified by the cooperation between Huawei and Seres, as well as Mobileye’s partnership with BMW, which has strengthened the manufacturer’s competitiveness through advanced autonomous driving solutions. Similarly, increased sensitivity to cost reduction encourages both vehicle manufacturers and core component suppliers to boost R&D investments, as demonstrated by the CATL-NIO cooperation on battery cost optimization. Such synergies not only reduce production costs but also enhance vehicle intelligence, as seen in the BMW-Bosch partnership in autonomous driving systems. Additionally, in the manufacturer-dominant model, a lower share of R&D costs borne by the manufacturer leads to increased R&D effort. With less financial burden, the manufacturer can invest more in product enhancements. In technology-dominated models, the optimal R&D effort is not influenced by the sensitivity of R&D cost reduction, which means core component suppliers prioritize product innovation over process improvements, with a focus on enhancing product quality. This is evident in the cooperation between Baidu Apollo and vehicle manufacturers, where Baidu Apollo’s R&D efforts focus on advancing the product’s intelligence rather than reducing production costs.

#### Proposition 2.

An increase in consumers’ sensitivity to product quality, coupled with a decrease in sensitivity to cost-reducing R&D, will lead to a rise in the vehicle’s price [21]. Additionally, in the technology-dominated model, vehicle prices remain consistently low. In the supplier cooperation model, a greater share of R&D costs borne by the vehicle manufacturer leads to higher prices. In the manufacturer-dominated model, a higher share of R&D costs borne by the vehicle manufacturer results in lower prices. Specifically,


$$\partial p_{SZ}^{*}/\partial \beta >0,\;\partial p_{ZH}^{*}/\partial \beta >0,\;\partial p_{LH}^{*}/\partial \beta >0;\;\partial p_{SZ}^{*}/\partial \theta <0,\;\partial p_{SZ}^{*}/\partial \tau <0,\;\partial p_{ZH}^{*}/\partial \tau >0.$$

Proposition 2 reveals the impact of consumers’ quality sensitivity, R&D cost-reduction sensitivity, and R&D cost-sharing on the vehicle’s price under different cooperation models. An increase in consumer sensitivity to product quality drives up vehicle prices, reflecting the heightened R&D investments required to meet this demand. For instance, companies like NIO and Tesla justify the premium pricing of models such as the ES8 and Model S by offering advanced features like autonomous driving and intelligent systems, which are the direct result of increased R&D efforts. Moreover, in the technology-dominated model, the optimal vehicle price is not influenced by either the consumer’s sensitivity to quality or the sensitivity of R&D to cost reduction. Combined with Proposition 1, this indicates that core component suppliers set lower prices to quickly capture market share, often without focusing on R&D-driven innovations. While this strategy boosts sales volume, it limits vehicle manufacturers’ profit margins, a dynamic observed in Waymo’s partnerships with vehicle manufacturers for autonomous driving technology. In the supplier-cooperation model, as the proportion of R&D costs borne by the manufacturer increases, the vehicle price also rises, and according to Proposition 1, the R&D effort level increases as well. For example, in the CATL-BMW partnership, CATL covers most battery R&D costs while BMW sets vehicle pricing. As BMW’s R&D share rises, the vehicle price increases, and the company invests more in intelligent features. Conversely, in the manufacturer-dominant model, a higher share of R&D costs borne by the manufacturer leads to a lower vehicle price, along with a decrease in R&D effort. For example, as BYD increased its proportion of in-house R&D, it reduced the level of intelligent features in certain models and set extremely low prices to remain competitive.

#### Proposition 3.

In the technology-dominated model, the core component supplier’s profit is independent of R&D cost reduction sensitivity but positively correlated with consumer sensitivity to product quality. The vehicle manufacturer’s profit is positively correlated with both. In the supplier-cooperation model, the core component supplier’s profit is positively correlated with both consumer sensitivity to product quality and R&D cost reduction sensitivity. Specifically, $\partial \prod_{SH}^{i*}/\partial \beta >0$, $\partial \prod_{SZ}^{S*}/\partial \beta >0$, $\partial \prod_{SH}^{Z*}/\partial \theta >0$, $\partial \prod_{SZ}^{S*}/\partial \theta >0$; $i\in \left\{S,Z\right\}$.

Proposition 3 illustrates how profits in the supply chain are shaped by consumer sensitivity to product quality and R&D cost reduction sensitivity. In the technology-dominant model, both the core component supplier and the vehicle manufacturer gain from heightened consumer sensitivity to product quality. However, only the vehicle manufacturer’s profit benefits from reductions in R&D costs. The core supplier’s profits, by contrast, depend more on market demand and product innovation than on cost reductions. Therefore, to maximize profits, core component suppliers increase consumers’ sensitivity to quality, which positively correlates with optimal R&D effort levels. As a result, the supply chain adopts a quality-focused strategy. A relevant example is the Huawei-Seres partnership, where Huawei’s profits depend on consumer demand for advanced technology, while Seres benefits from reduced production costs. Furthermore, in the supplier-cooperation model, heightened sensitivity to both product quality and R&D cost reduction positively impacts the core supplier’s profits. Consequently, core component suppliers enhance both factors, each correlating positively with the supply chain’s optimal R&D effort level, leading the supply chain to at least adopt a quality-focused strategy. An example is the partnership between General Motors and LG Chem, where LG Chem enhances battery quality while reducing costs, allowing GM to adopt a quality-focused strategy to attract consumers seeking advanced technology.

### 5.2 Comparative analysis of cooperative research and development models

#### Proposition 4.

The relative degree of product and process innovation will influence the optimal R&D effort across different cooperative R&D models, thereby demonstrating shifts in supply chain power as the relative magnitudes of these two factors change. Specifically, when β>bθ, $e_{SH}^{*}>e_{SZ}^{*}$;when β<bθ, $e_{SH}^{*}<e_{SZ}^{*}$;when k=0 and τ=0, $e_{LH}^{*}>e_{ZH}^{*}$.

Proposition 4 reveals that the balance between product and process innovation in different supply chain models impacts the optimal R&D effort. When product innovation surpasses process innovation, pricing authority shifts from the vehicle manufacturer to the core component supplier, moving from a supplier cooperation model to a technology-dominated model. For example, in the Huawei-Seres partnership, Huawei leads R&D and pricing due to its focus on product innovation, favoring a quality strategy. Conversely, when process innovation takes precedence, pricing authority returns to the vehicle manufacturer, as seen in the Waymo-FCA partnership, where production optimization left pricing control with FCA. In this case, the supplier cooperation model achieves higher R&D effort, especially in cost reduction and process innovation. In terms of R&D costs, if these costs are fully transferred to the vehicle manufacturers, the joint decision-making model yields in higher R&D effort compared to the manufacturer-dominant model. This is because, in this scenario, core component suppliers bear almost no R&D costs, incentivizing them to increase their R&D investment for higher profits, and resulting in greater R&D effort than the manufacturer-dominant model. Although this represents an extreme scenario unlikely to occur in commercial practice, it nonetheless highlights this trend.

#### Proposition 5.

Technology-dominant vehicles sell for the lowest price.

Proposition 5 reveals that in the technology-dominant model, where the core component supplier leads, the supply chain prioritizes market share over short-term profits. Companies like Tesla and BYD adopt low-price strategies primarily for two reasons: First, lower prices drive sales volume and market share, leveraging economies of scale to reduce costs [9]. This approach supports future revenue through software-based services, as ‘software-defined vehicles’ generate recurring income from updates, subscriptions, and add-on features. Second, capturing market share enhances a company’s brand position, which can strengthen investor confidence and positively impact stock prices. However, a technology-dominant model may also risk triggering price wars, where the pricing power of component suppliers pressures vehicle manufacturers into adopting sustained low-price strategies. This approach requires careful balance to maintain profitability and long-term growth in a highly competitive market.

### 5.3 Analysis of influencing factors on the optimal R&D effort and vehicle price

#### Proposition 6.

The relative contribution of the consumer quality sensitivity coefficient and R&D cost reduction sensitivity coefficient to optimal R&D effort is determined by the consumer price sensitivity coefficient. Specifically, by setting $f\left(\beta ,\theta \right)=e_{j}^{*}$, $j\in \left\{SZ,ZH,LH\right\}$, we obtain $\left|f^{'}{\left(\beta ,\theta \right)}_{\theta }/f^{'}{\left(\beta ,\theta \right)}_{\beta }\right|=b$.

Proposition 6 reveals that as consumer price sensitivity increases, the contribution of R&D cost reduction to the optimal R&D effort surpasses that of quality sensitivity. Conversely, as price sensitivity decreases, quality sensitivity becomes more influential. When consumers prioritize price, cost reduction becomes the key strategy for the supply chain. Lowering prices enhances competitiveness, attracting price-sensitive buyers. On the other hand, when price sensitivity is low, consumers focus on quality. In this case, vehicle manufacturers prioritize enhancing vehicle features.

#### Proposition 7.

Under the supplier cooperation model, the relative contribution of the consumer quality sensitivity coefficient and R&D cost sensitivity coefficient to the optimal product price is affected by consumer preference and R&D cost. Specifically, by setting $l\left(\beta ,\theta \right)=p_{SZ}^{*}$, we obtain:


$$\left|l^{'}{\left(\beta ,\theta \right)}_{\theta }/l^{'}{\left(\beta ,\theta \right)}_{\beta }\right|=b\left(\left(w-g\right)-k\right)/\left(\left(w-g\right)+k\right)$$

Proposition 7 reveals that as consumer price sensitivity increases or R&D costs decrease, the contribution of cost reduction to optimal pricing surpasses that of quality sensitivity. Conversely, when price sensitivity decreases or R&D costs rise, quality sensitivity plays a larger role in determining prices. When price sensitivity is high, R&D efforts to reduce component costs allow vehicle manufacturers to lower vehicle prices, making cost reduction a key driver of pricing. Conversely, when consumers prioritize quality over price, vehicle manufacturers focus on product enhancements and set higher prices to meet demand.

## 6 Numerical analysis

To improve the readability of the propositions and inferences, this paper utilizes Python for numerical analysis to verify the impact of various parameters on R&D effort, vehicle pricing, and total profits of the players under the four models. Specifically, this paper utilizes Python version 3.11.5, with the execution environment provided by Anaconda version 23.7.4 on the macOS (osx-arm64) platform. All code is executed in the base environment of Anaconda to ensure consistency in library versions and stability during runtime. The implementation primarily employs the following libraries: NumPy for efficient numerical computation and matrix operations, Matplotlib and mpl_toolkits.mplot3d for two-dimensional and three-dimensional plotting and result visualization, and Matplotlib rcParams for configuring the display style and parameters of the plots. These tools collectively facilitate data processing, analysis, and visualization. The data, which meet the relevant assumptions and constraints, are based on the studies by Zheng YL et al. [6] and the Global EV Outlook report from the International Energy Agency (IEA) [49]. Setting a=5000, b=1.2, h=1000, k=100, w=1000, g=800, $c_{0}=2000$, $p_{0}=1500$. Unless otherwise specified, the effect of one parameter is analyzed assuming that the other parameters remain unchanged.

### 6.1 Comparison of R&D effort and analysis of influencing factors

**Fig 2:**
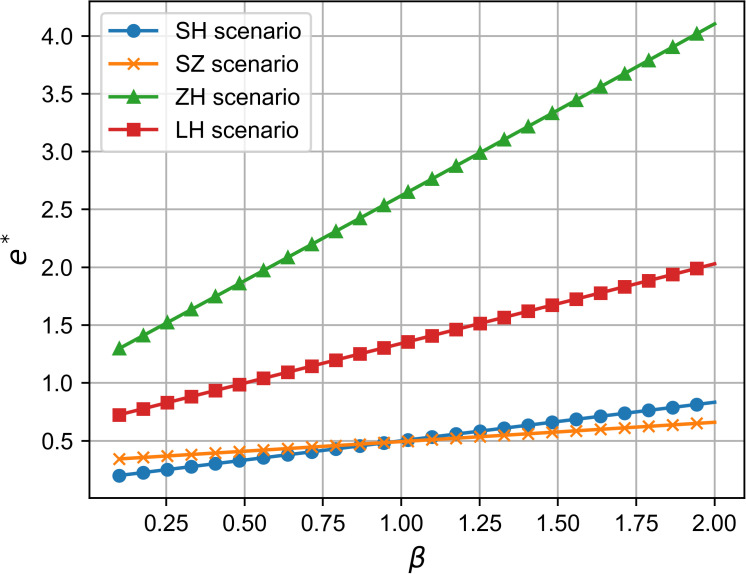
The influence of β on e^*^.

**Fig 3:**
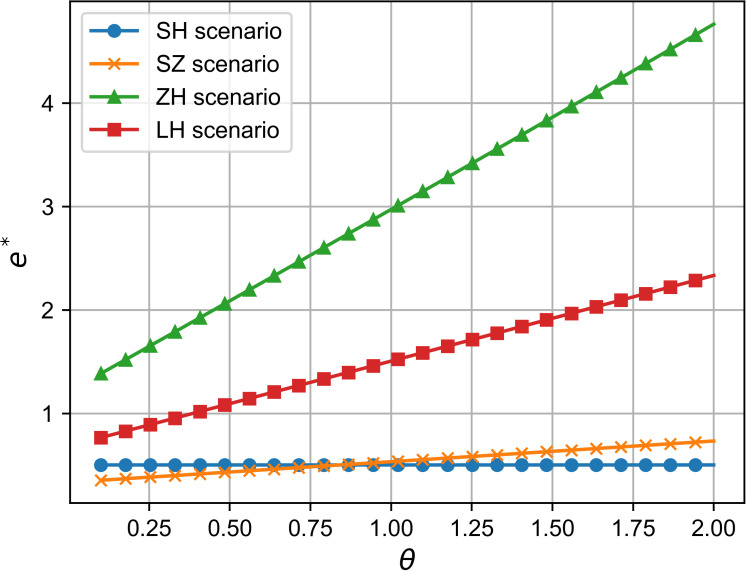
The influence of θ on e^*^.

As shown in Figs 2 and 3, across all four cooperation models, the optimal R&D effort increases with both the quality sensitivity coefficient and the product R&D cost reduction sensitivity coefficient, indicating a positive correlation between these coefficients and R&D investment. Whether improving product quality or reducing R&D costs, both drive firms to boost R&D efforts to stay competitive, supporting Proposition 1. Moreover, the simulation results in Figs 2 and 3 show that the impact of consumers’ quality sensitivity coefficient and the R&D cost-reduction sensitivity coefficient on the optimal R&D effort is consistent [21]. In the manufacturer-dominant model, the two coefficients have the greatest impact on the optimal R&D effort level, and the optimal R&D effort level is the highest at this point. Additionally, Figs 2 and 3 reveal that the technology-dominated model surpasses the supplier cooperation model in terms of optimal R&D effort when consumer quality sensitivity and R&D cost reduction sensitivity are high. This suggests that in the technology-dominated model, core component suppliers are more likely to increase R&D efforts compared to the supplier cooperation model when these factors are critical. According to the simulation results, if the cooperative R&D in the supply chain aims for a high level of intelligence, the manufacturer-dominant model is a more suitable choice.

**Fig 4:**
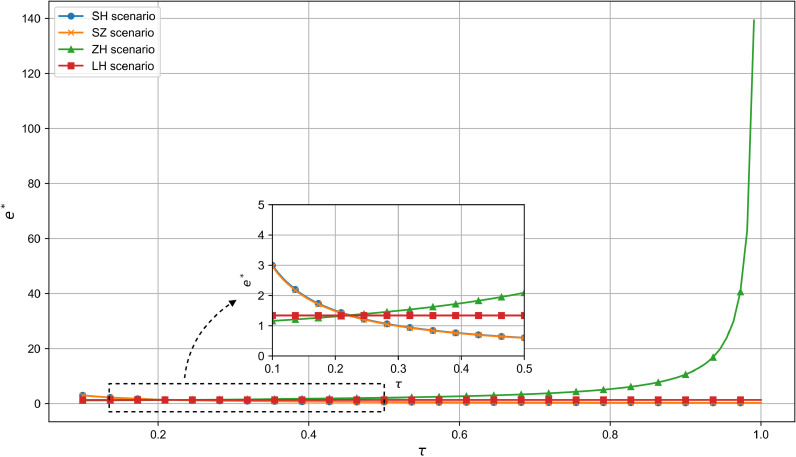
The influence of τ on e^*^.

As shown in Fig 4, the optimal R&D effort in the technology-dominated and supplier cooperation models decreases as core component suppliers bear a larger share of R&D costs. In contrast, the joint decision-making model remains unaffected. In the manufacturer-dominated model, the lower the proportion of R&D costs borne by the manufacturer, the greater the R&D effort. In the joint decision-making model, R&D effort remains stable regardless of cost-sharing, ensuring balanced cooperation. This highlights how supply chain participants optimize resource allocation for efficiency and long-term cooperation, supporting Proposition 1.

### 6.2 Comparison of vehicle selling price and analysis of influencing factors

**Fig 5:**
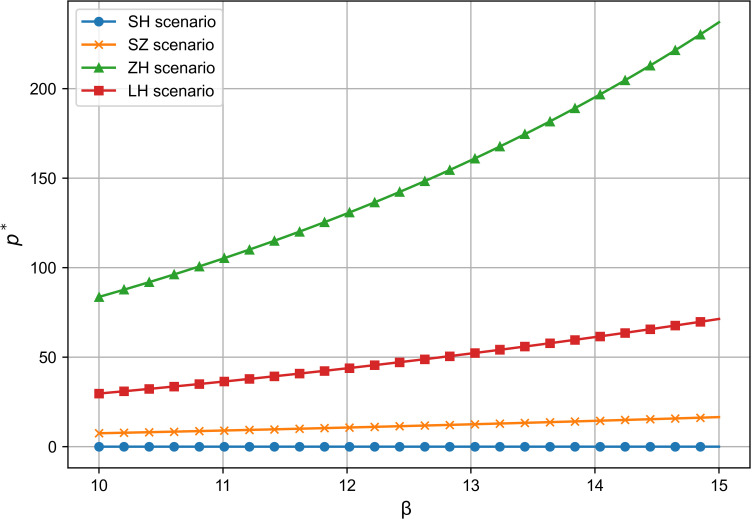
The influence of β on p^*^.

**Fig 6:**
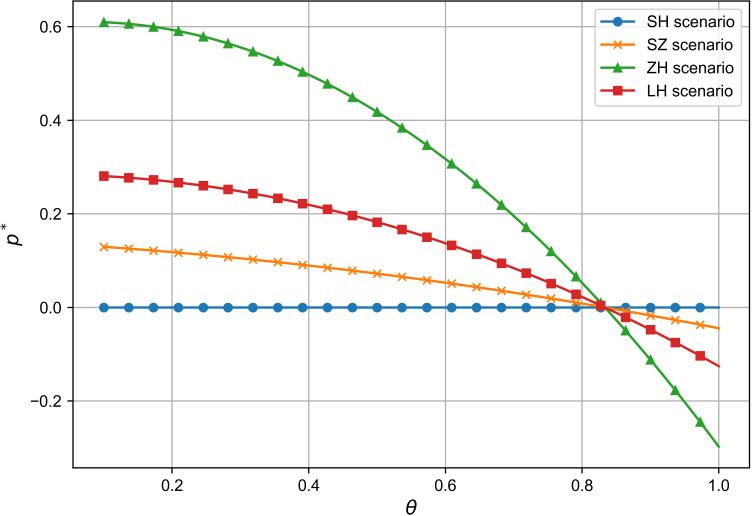
The influence of θ on p^*^.

As shown in Figs 5 and 6, due to the minimal impact of the quality sensitivity coefficient and the R&D cost reduction sensitivity coefficient on vehicle pricing, the base price is set to zero in the simulation in Section 6.2 for clearer observation of these correlations. In all models except the technology-dominated model, vehicle prices rise as these coefficients increase, supporting Propositions 2 and 5. Additionally, the results show a negative correlation between the R&D cost reduction sensitivity and vehicle pricing: as R&D reduces production costs, prices decrease to remain competitive, aligning with Proposition 2. However, the overall effect of both coefficients on pricing remains small.

**Fig 7:**
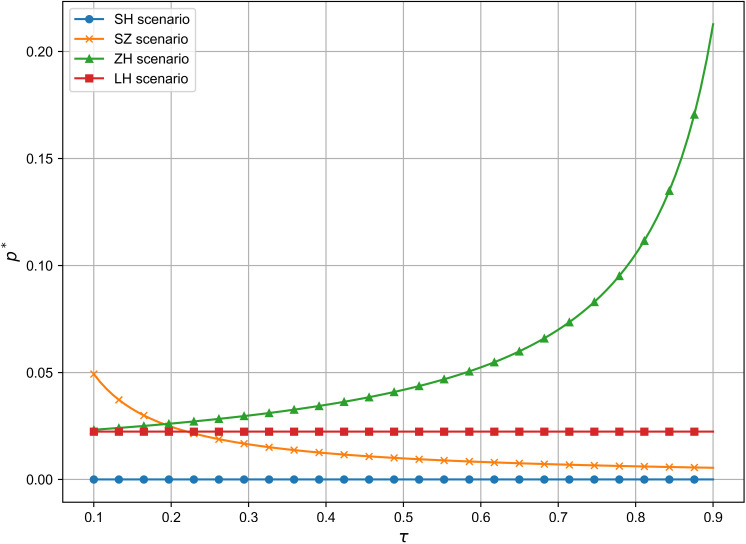
The influence of τ on p^*^.

As shown in Fig 7, vehicle pricing is independent of the manufacturer’s R&D cost share in the technology-dominated and joint decision-making models. However, in the supplier cooperation model, there is a negative correlation between vehicle pricing and the core component suppliers’ R&D share, while in the manufacturer-dominated model, this correlation is positive. This reflects that in the manufacturer-dominated model, where the manufacturer controls R&D decisions, prices rise as core component suppliers take on more R&D costs, consistent with Proposition 2. In addition, while the manufacturer’s R&D share impacts pricing in some models, the overall effect is limited. This suggests that factors like market competition, consumer demand, and economies of scale are more influential in determining vehicle pricing than the R&D cost share. For instance, Toyota’s Prius pricing strategy remained stable despite significant R&D investment, as economies of scale played a larger role in cost management.

### 6.3 Profit comparison and analysis of influencing factors

**Fig 8:**
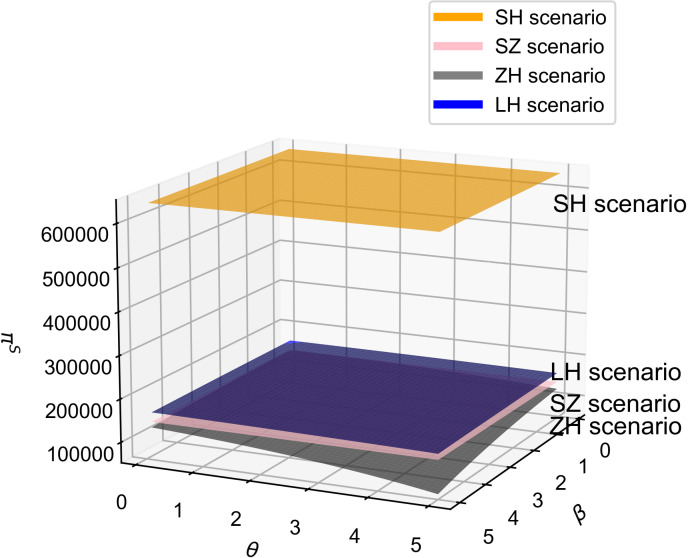
The influence of β and θ on Π^s^.

As shown in Fig 8, the impact of consumer quality sensitivity and R&D cost reduction sensitivity on core component supplier profits varies by cooperation model. For core component suppliers, profits are highest in the technology-dominant model. Therefore, when selecting a cooperative model, core component suppliers should aim to establish themselves as the industry’s key players and adopt a technology-dominant model; if not a core player, the joint decision-making model is preferable. Additionally, in the technology-dominant model, the core component supplier’s profit increases with the consumer quality sensitivity coefficient. Acting as the dominant player, the supplier may use promotional strategies to elevate this coefficient to maximize profits, as it positively correlates with R&D effort. Consequently, the supply chain adopts a quality-focused strategy, consistent with Proposition 3.

**Fig 9:**
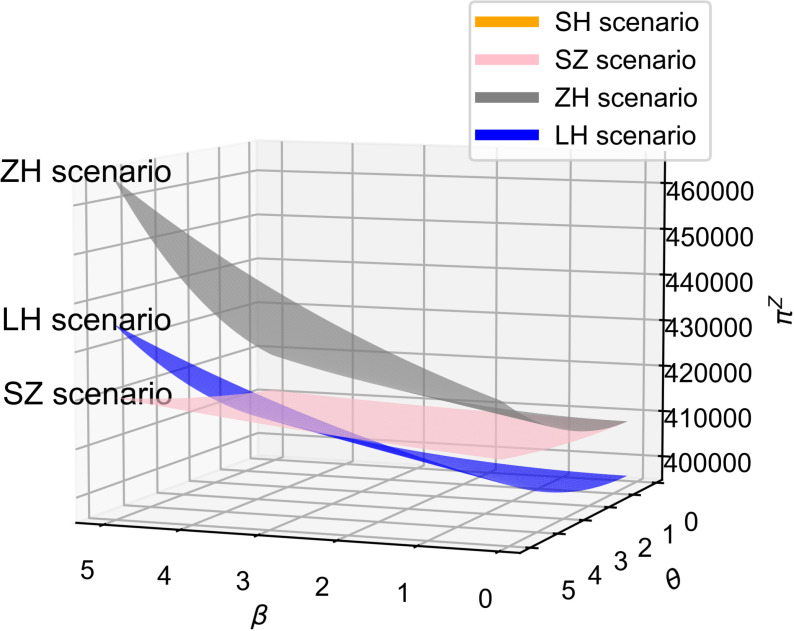
The influence of β and θ on Π^z^.

As shown in Fig 9, vehicle manufacturer profits increase in the supplier-cooperation, manufacturer-dominant, and joint decision-making models as consumer quality sensitivity and R&D cost reduction sensitivity rise, aligning with Proposition 3. Additionally, vehicle manufacturer profits are highest in the manufacturer-dominant model [22–24]. Therefore, when choosing a cooperative model, vehicle manufacturers should aim to establish themselves as industry leaders and select the manufacturer-dominant model; if they are not key players, they should consider the joint decision-making model in highly competitive markets or the supplier-cooperation model when competition is less intense. With an increase in consumer quality sensitivity and R&D cost reduction sensitivity, profits under the joint decision model will exceed those in the supplier-cooperation model. It is also worth noting that, under this simulation, profits in the technology-dominant model turned negative, which is why it is not shown in Fig 9, suggesting that the technology-dominant model may be unfavorable for vehicle manufacturers.

Additionally, based on Proposition 3, Figs 8 and 9, we observe that in the supplier-cooperation model, the profits of both core component suppliers and vehicle manufacturers are positively correlated with consumer quality sensitivity and R&D cost reduction sensitivity. Consequently, the supply chain strives to increase these two coefficients, which also positively correlate with R&D effort levels, leading the supply chain to adopt a quality-focused strategy in the supplier-cooperation model. However, these two coefficients have opposing effects on vehicle pricing: one positive and one negative. According to Proposition 9, when consumer price sensitivity is high, the impact of R&D cost reduction sensitivity surpasses that of quality sensitivity. In summary, in the supplier-cooperation model, the supply chain adopts a quality-focused strategy and, as consumer price sensitivity rises, simultaneously pursues a low-price strategy.

**Fig 10:**
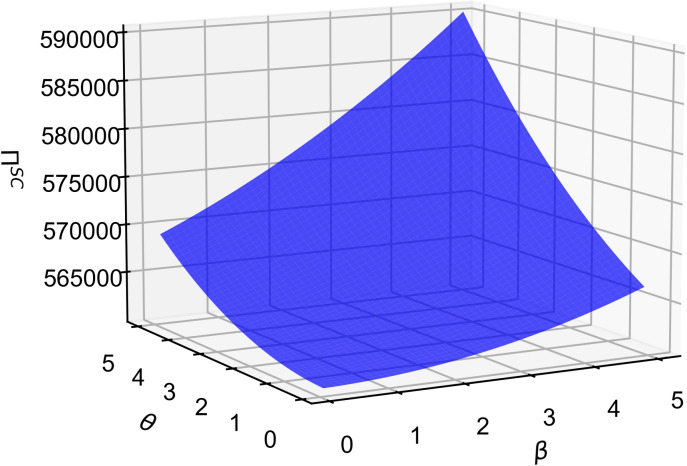
The influence of β and θ on Π^sc^.

As shown in Fig 10, supply chain profits in the joint decision-making model increase with higher consumer quality and R&D cost reduction sensitivity. Combining Figs 8 and 9, these coefficients have opposing effects on the core component supplier and vehicle manufacturer: they positively correlate with manufacturer profits but negatively with supplier profits. From a supply chain power structure perspective, the manufacturer typically holds a dominant position in the joint decision-making model, allowing it to shift the pressure of quality sensitivity and cost control onto the supplier, who then bears more costs to enhance product quality. This indicates that the resulting sales growth and market expansion from these coefficients more than offset the supplier’s reduced profits, thus boosting overall supply chain profitability.

**Fig 11:**
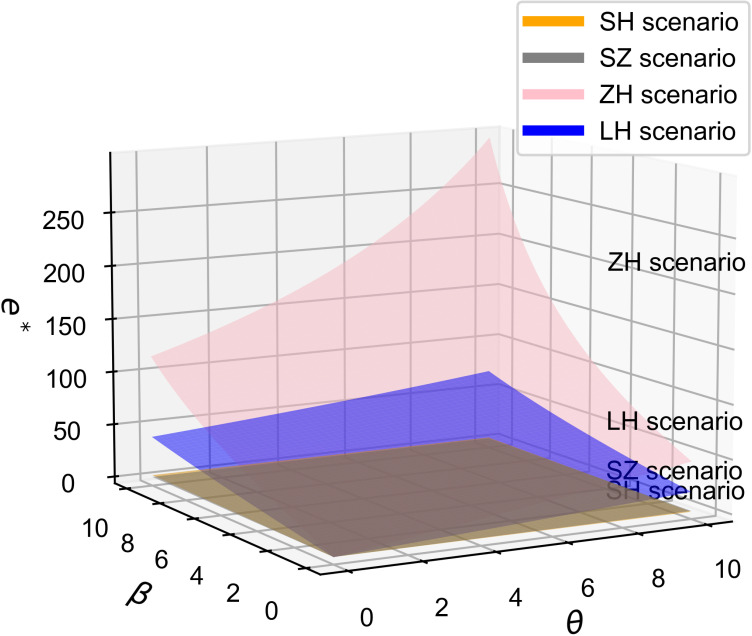
The influence of β and θ on e^*^, b = 0.5.

**Fig 12:**
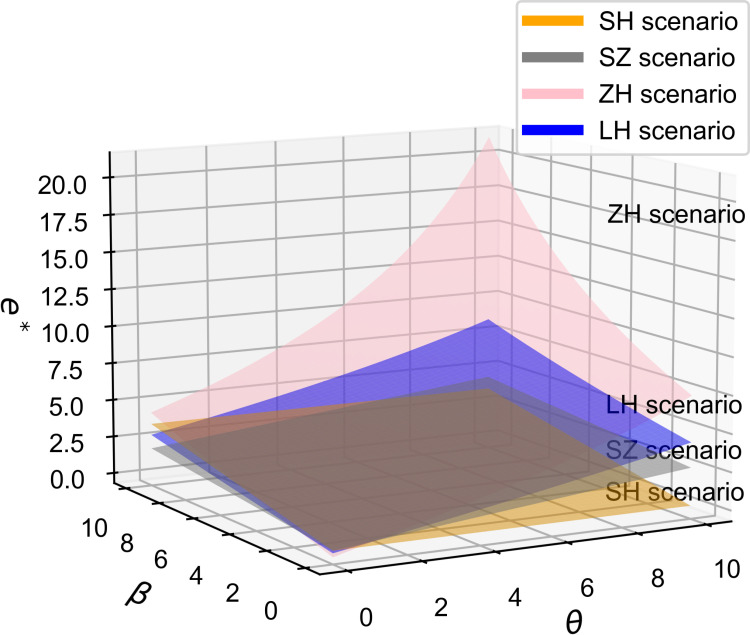
The influence of β and θ on e^*^, b = 1.5.

As shown in Figs 11 and 12, with the increase in the consumer price sensitivity coefficient, moving from Fig 11 to Fig 12, it can be observed that the influence of the consumer quality sensitivity coefficient on the optimal R&D effort decreases, while the influence of the R&D cost reduction sensitivity coefficient increases. This is in line with Proposition 6.

**Fig 13:**
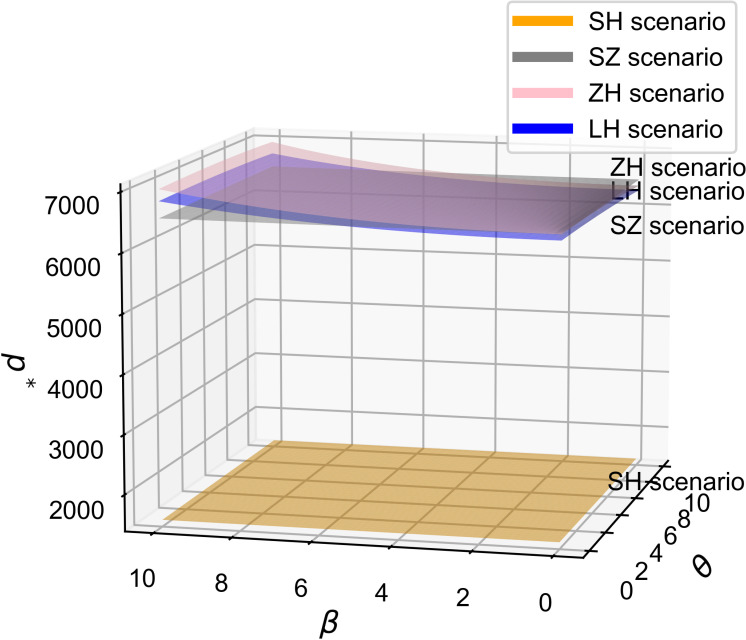
The influence of β and θ on p^*^, b = 0.5.

**Fig 14:**
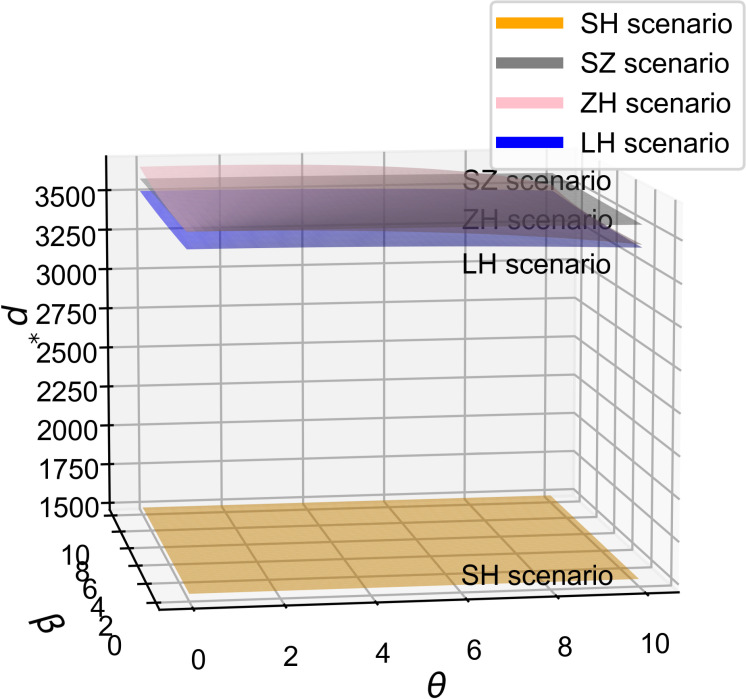
The influence of β and θ on p^*^, b = 1.2.

As shown in Figs 13 and 14, as the consumer price sensitivity coefficient increases, moving from Fig 13 to Fig 14, the influence of the consumer quality sensitivity coefficient on the optimal vehicle price diminishes, while the influence of the R&D cost reduction sensitivity coefficient grows. This observation aligns with Proposition 7. Additionally, based on Figs 8, 9, and 11, in the manufacturer-dominant model, manufacturer profits positively correlate with both coefficients, even though supplier profits are negatively affected. Since the manufacturer is the dominant player, the supply chain adopts a quality-focused strategy and, when consumer price sensitivity is high, also implements a low-price strategy. In the joint decision-making model, supply chain profits positively correlate with both parameters; thus, similarly, the supply chain adopts a quality-focused strategy and adjusts the low-price strategy according to the level of consumer price sensitivity.

## 7 Conclusion

### 7.1 Discussion

In response to the current R&D and price competition under cross-organizational cooperative models in the intelligent vehicle supply chain, this paper proposes four cooperative R&D models. The aim is to analyze the applicability conditions of different models, the shifts in supply chain power across models [6–9], and the strategic choices under each model [46], with a focus on the impact of product innovation and process innovation on R&D effort and pricing decisions. By analyzing and comparing the equilibrium outcomes of core component suppliers and vehicle manufacturers across the four models and evaluating the impact of various parameters on these equilibrium results, the following key conclusions are drawn.

First, we analyze the conditions under which different cross-organizational R&D cooperation models are applicable and the shifts in power structures associated with each model. We find that the profits of both core component suppliers and vehicle manufacturers are higher in models where they hold a dominant position compared to non-dominant positions [6, 22–24]. Therefore, when selecting a cooperative model, core component suppliers should choose the technology-dominant model, while vehicle manufacturers should opt for the manufacturer-dominant model. If not in a leading position, the joint decision-making model is more appropriate for both core component suppliers and vehicle manufacturers. Additionally, if the supply chain aims to maximize vehicle intelligence rather than short-term profits, the manufacturer-dominant model is preferable. This paper also reveals that when product innovation from R&D exceeds process innovation, pricing power shifts from vehicle manufacturers to core component suppliers, transitioning the supply chain from the supplier-cooperation model to the technology-dominant model. Conversely, when process innovation surpasses product innovation, pricing power reverts to vehicle manufacturers, shifting the supply chain from the technology-dominant model to the supplier-cooperation model.

Second, we analyze the strategic choices across different supply chain power structures. In the technology-dominant model, the supply chain prioritizes a quality-focused strategy. Although this may introduce a risk of price wars, it is not an intentional outcome. In contrast, in the supplier-cooperation model, the manufacturer-dominant model, and the joint decision-making model, the supply chain consistently adopts a quality strategy. However, the decision to implement a low-price strategy alongside the quality strategy depends on consumer price sensitivity. As consumer price sensitivity increases, the impact of the R&D cost reduction coefficient on vehicle pricing grows stronger than that of the product innovation coefficient, thereby making the cost-reduction effect more significant than the price increase driven by product innovation. Thus, as consumer price sensitivity increases, the likelihood of the supply chain simultaneously adopting both quality and low-price strategies rises. Conversely, when consumer price sensitivity is lower, the supply chain tends to rely solely on the quality strategy.

Finally, we examine the impact of key parameters on the equilibrium outcomes. We find consistent positive effects of process and product innovation coefficients on the optimal R&D effort level across various supply chain power structures [21]. However, while the process innovation coefficient is negatively correlated with the optimal vehicle price, the product innovation coefficient shows a positive correlation. A notable distinction appears in the manufacturer-dominated model, where both the optimal R&D effort and vehicle price are negatively correlated with the manufacturer’s R&D cost-sharing ratio. In contrast, in other power structures, these metrics are positively correlated with the manufacturer’s R&D cost-sharing ratio. The technology-dominant model consistently maintains the lowest optimal vehicle price, which remains unaffected by the product or process innovation coefficients or cost-sharing ratios. Simulation results further indicate that the manufacturer-dominant model achieves the highest level of product intelligence [5]. Additionally, as consumer price sensitivity rises, the influence of the R&D cost reduction coefficient on both R&D effort and vehicle price surpasses that of the quality sensitivity coefficient.

### 7.2 Management implications

Against the backdrop of high R&D costs in vehicle intelligence, this paper aims to explore the applicability of different supply chain cooperation models, power shifts, and strategic choices, providing valuable insights for technology companies and vehicle manufacturers on cooperation model selection, strategic choices, and R&D effort and pricing decisions within the context of cross-industry joint R&D in the intelligent vehicle supply chain. Specifically, it offers the following insights for participants in the intelligent vehicle supply chain.

First, companies should select cooperation models flexibly based on supply chain positioning and competitive advantages [8,15]. Since profits in a dominant position are consistently higher than those in a non-dominant role [6,22–24]. For instance, core component suppliers like Huawei or Google may benefit from a technology-dominant model to control R&D and innovation, while core vehicle manufacturers such as Tesla and BYD are better suited to a manufacturer-dominant model, enabling control over vehicle intelligence and cost management [16]. If neither party holds a core position, as with startups in emerging markets, a joint decision-making model allows resource sharing for mutual benefit. When cross-industry R&D has strategic long-term importance, such as developing advanced intelligence to establish competitive barriers, the manufacturer-dominant model is preferable. Additionally, the high technological demands of the intelligent vehicle industry make cooperation essential, with partnerships among vehicle manufacturers, tech firms, and data companies helping to complement resources and drive innovation. Such partnerships enable vehicle manufacturers to accelerate intelligent technology advancements, while component suppliers can leverage alliances with AI and autonomous driving firms to maintain their technological edge.

Second, intelligent vehicle companies should first leverage AI and data mining to deeply analyze consumer preferences, market dynamics, and technological trends, providing foundational support for strategic choices [23,42]. In different cooperation models, companies should adapt their strategies based on consumer quality and price sensitivity. In the technology-dominant model, a quality-focused strategy is recommended for the supply chain, although the potential risk of price wars should be anticipated and mitigated through differentiation and brand premium strategies. In the supplier-cooperation, manufacturer-dominant, and joint decision-making models, quality remains the core strategy, but a low-price strategy should be integrated if consumer price sensitivity is high to enhance market competitiveness. In these models, companies need to balance product quality enhancement with cost optimization to maximize profitability [44]. Furthermore, as consumer price sensitivity rises, companies should emphasize cost optimization through cross-organizational cooperation and process innovation, effectively sharing R&D cost pressures. This approach helps achieve an optimal balance between quality and cost, ensuring the sustainability and adaptability of strategic choices in a competitive market.

Finally, based on the research findings, companies in the intelligent vehicle supply chain should align their R&D and pricing strategies with their innovation priorities and supply chain power structures [23–25]. Specifically, intelligent vehicle supply chain companies should optimize R&D resource allocation by balancing process and product innovation to enhance product quality while controlling costs. In the manufacturer-dominant model, manufacturers can reduce their share of R&D costs to drive product intelligence and maintain a pricing advantage. Conversely, in supplier-cooperation and joint decision-making models, moderately increasing the cost-sharing ratio can help sustain innovation investment. In the technology-dominant model, the supply chain may adopt a low-price strategy to capture market share, particularly in price-sensitive markets. As consumer price sensitivity rises, companies should focus on process innovation and cost control, employing data analysis to dynamically monitor consumer price sensitivity and ensure that R&D direction and resource allocation align with market demands [24,42]. This data-driven management approach not only supports competitive pricing but also enables a balanced focus on product intelligence and cost optimization, thereby enhancing adaptability and long-term competitiveness in a rapidly changing market.

### 7.3 Limitations and future research

This paper can be further expanded and enriched in several ways. First, this paper treated the wholesale price of components as an exogenous variable, but future research could explore endogenous wholesale prices by examining the differences between joint R&D and independent R&D, especially linking wholesale prices to order volumes. Particularly for the procurement of high-cost intelligent components in intelligent vehicles, varying demand levels and different R&D cooperation models may lead to different wholesale pricing strategies. Second, while this paper focused on wholesale pricing contracts, real-world cooperation between core component suppliers and vehicle manufacturers often involves joint design, R&D, sales, and services, where revenue-sharing contracts are more common [46]. Investigating these contracts could provide valuable insights into alternative pricing mechanisms. Third, this paper only examined strategic choices in a single supply chain, but competition between different supply chain cooperation models also exists. Future research could incorporate these competitive dynamics to provide a more comprehensive understanding of the cross-industry R&D landscape. Lastly, previous studies by Yan et al. [11] and Wu et al. [12] suggest that privileged royalty licensing can effectively mitigate price competition and enable higher profit margins. Future research could explore how similar mechanisms might be applied to cooperative R&D to reduce price competition and enhance profitability across the supply chain.

## Supporting information

S1 Data Dataset.
(ZIP)
