# Supplementary material for: R&D and pricing strategies in the intelligent vehicle supply chain under cross-organizational cooperative models
Source: PLoS One. 2025 Apr 24;20(4):e0321903. doi: 10.1371/journal.pone.0321903 (PMC12021157; doi:10.1371/journal.pone.0321903)
Supplement: S1 Data — Dataset. (ZIP) [file pone.0321903.s001.zip › S1_Dataset/Minimal data set.docx]

**Minimal data set**

This paper first validates, through mathematical modeling, the impact of the process innovation coefficient, product innovation coefficient, and research and development (R&D) cost-sharing ratio on the optimal R&D effort level, optimal vehicle price, and the optimal profits of core component suppliers and vehicle manufacturers under four cooperative modes. All computational procedures involved in this paper are presented in the manuscript, and readers can verify our results by following the detailed calculations.

To improve the readability of the propositions and inferences, this paper utilizes Python for numerical analysis to verify the impact of various parameters on R&D effort, vehicle pricing, and total profits of the players under the four models. Specifically, this paper utilizes Python version 3.11.5, with the execution environment provided by Anaconda version 23.7.4 on the macOS (osx-arm64) platform. All code is executed in the base environment of Anaconda to ensure consistency in library versions and stability during runtime. The implementation primarily employs the following libraries: NumPy for efficient numerical computation and matrix operations, Matplotlib and mpl_toolkits.mplot3d for two-dimensional and three-dimensional plotting and result visualization, and Matplotlib rcParams for configuring the display style and parameters of the plots. These tools collectively facilitate data processing, analysis, and visualization.

In numerical analysis of this paper, the original data of parameters are:, , , , , , , . Unless otherwise specified, the effect of one parameter is analyzed assuming that the other parameters remain unchanged. It is also necessary to note that Figs 11 and 12 in the manuscript provide a separate comparative analysis of the consumer price sensitivity coefficient. Specifically, in Fig 11, Fig. 11(a) corresponds to and Fig. 11(b) corresponds to , while in Fig 12, Fig. 12(a) corresponds to and Fig. 12(b) corresponds to . The plotting of these figures is based on the four theorems presented in the manuscript. And all the specific graph-generating python code is also provided in the support information.
